# Performance and Egg Quality of Laying Hens Fed Diets Containing Raw, Hydrobarothermally-Treated and Fermented Rapeseed Cake

**DOI:** 10.3390/ani11113083

**Published:** 2021-10-28

**Authors:** Magdalena Kopacz, Aleksandra Alicja Drażbo, Katarzyna Śmiecińska, Katarzyna Ognik

**Affiliations:** 1 Department of Poultry Science and Apiculture, University of Warmia and Mazury in Olsztyn, Oczapowskiego 5, 10-719 Olsztyn, Poland; magdalena.kopacz@uwm.edu.pl; 2 Department of Commodity Science and Animal Raw Material Processing, University of Warmia and Mazury in Olsztyn, Oczapowskiego 5, 10-719 Olsztyn, Poland; katarzyna.smiecinska@uwm.edu.pl; 3 Department of Biochemistry and Toxicology, Faculty of Animal Sciences and Bioeconomy, University of Life Sciences in Lublin, Akademicka 13, 20-950 Lublin, Poland; katarzyna.ognik@up.lublin.pl

**Keywords:** laying hen, nutrition, egg quality, rapeseed cake, fermentation, hydrobarothermal treatment

## Abstract

**Simple Summary:**

Rapeseed cake (RC) has recently gained increasing interest as a dietary protein source alternative to soybean meal (SBM). However, its wider use in poultry diets (including laying hen diets) is limited due to the high concentrations of antinutritional compounds. Technological processes such as thermal treatment and fermentation may improve the nutritional value of RC by reducing the content of non-starch polysaccharides (NSPs), glucosinolates (GLS) and phytate phosphorus (PP). The present study revealed that the inclusion of 20% RC in layer diets improves the fatty acid (FA) profile of egg yolks while maintaining a desirable redox status. Hydrobarothermally-treated RC (HRC) and fermented RC (FRC) exerted a greater beneficial influence on the laying performance of hens than raw RC (RRC). FCR appears to be the optimal substitute for SBM because it contributed to the highest albumen quality and the highest sensory quality of eggs.

**Abstract:**

The present study was conducted to investigate how raw rapeseed cake (RRC), hydrobarothermally-treated rapeseed cake (HRC) and fermented rapeseed cake (FRC) fed to laying hens over a period of 12 weeks affected their performance, and the quality, fatty acid (FA) profile and oxidative stability of eggs. A total of 304 Hy-Line Brown laying hens at 36 weeks of age were distributed in a completely randomized design to four treatment groups with 38 replicates per treatment and two hens per replicate. The birds had ad libitum access to feed and water throughout the study. During the experiment, the birds were fed isonitrogenous and isocaloric diets in mash form, with various protein sources. In the control group (C), soybean meal (SBM) was the main source of dietary protein, whereas the experimental groups were fed diets containing 20% of RRC, HRC or FRC. Hydrobarothermal treatment and fermentation decreased the glucosinolate (GLS) content of RC, and fermentation reduced the concentration of phytate phosphorus (PP). In comparison with the RRC group, layers from the HRC and FRC groups were characterized by higher laying performance, comparable with that in group C. Irrespective of its physical form, RC added to layer diets adversely affected eggshell quality in all experimental groups, whereas albumen quality was highest in the FRC group. In comparison with group C, diets containing RRC, HRC and HRC led to a significant decrease in the content of saturated fatty acids (SFAs), an increase in the proportion of n-3 and n-6 polyunsaturated fatty acids (PUFAs) in the total FA pool in egg yolks, and a decrease in the n-6/n-3 PUFA ratio. The inclusion of RRC, HRC and FRC in layer diets decreased the activity of superoxide dismutase (SOD) in egg yolks, relative to group C. Group FCR eggs were characterized by the highest activity of catalase (CAT) and the lowest lipid peroxides LOOH concentration, compared with the remaining groups. The addition of RC to layer diets did not compromise the sensory quality of eggs, and eggs produced in group FRC received the highest overall score. It can be concluded that the inclusion of 20% RRC, HRC and FRC in layer diets does not compromise the sensory quality of eggs and has a beneficial influence on the FA profile and antioxidant potential of egg yolks. The use of FRC is recommended because it contributes to the highest laying performance, superior albumen quality and the highest sensory quality of eggs, relative to RRC and HRC.

## 1. Introduction

Soybean meal (SBM) is presently the main source of protein in animal diets, including laying hen diets. However, the global demand for feed protein is met by SBM imports from a limited number of countries. Due to the high prices and variable supply of SBM, as well as concerns over genetically modified soybeans, alternative domestic sources of vegetable protein, such as rapeseed meal (RSM) or rapeseed cake (RC), are being sought.

According to Food and Agriculture Organization (FAO) [1], in 2019/2020, global oilseed production reached 584.3 million tons, including 69.2 million tons of rapeseed. Rapeseed is widely used in the production of both foodstuffs and feedstuffs. Rapeseed oil by-products are utilized in the feed industry. These by-products include RC which can be a valuable component in layer diets [2]. The production technology of RC is less expensive and more environmentally friendly than organic solvent extraction [3]. RC is also a richer source of oil and metabolizable energy for poultry than RSM. Rapeseed oil is abundant in omega-3 (approx. 8–9%) and omega-6 (approx. 20–24%) polyunsaturated fatty acids (PUFAs), and it can increase the content of these fatty acids in the egg yolk [4,5]. Rapeseed oil also contains bioactive compounds, including vitamin E, phenolic compounds, flavonoids, phytosterols and other antioxidants that deliver health benefits for humans and animals [6].

The high content of non-starch polysaccharides (NSPs), glucosinolates (GLS) and phytic acid (PA) limits the use of RC in layer diets. Indigestible NSPs compromise the utilization of nutrients, in particular protein, energy and phosphorus [7]. Phytic acid is poorly available to poultry, therefore poultry diets have to be supplemented with phytase enzymes or inorganic phosphorus [8,9]. Glucosinolates, the major antinutritional compounds in rapeseed, increase mortality and decrease egg production and egg weight [10]. Some GLS degradation products contribute to the production of fish-tainted eggs whose yolks contain high levels of trimethylamine (TMA), a product of bacterial fermentation of choline in the lower gut [11]. Goitrin, an antinutritional factor (ANF) formed by the action of myrosinase on GLS, inhibits the oxidation of TMA to odorless compounds, which produces a fishy taint in eggs laid by genetically susceptible birds, specifically brown layers [12,13]. In the literature, there is no consensus on the optimal proportions of rapeseed products in layer diets. According to some authors, the rapeseed content of layer diets should not exceed 10%, whereas in other studies, diets containing 20% of rapeseed had no negative effect on laying performance or egg quality [14,15,16,17,18].

The nutritional value of rapeseed can be improved by reducing the content of some ANFs, which can be accomplished through physical, chemical and biological treatment or by modifying crop breeding practices [19,20,21]. Numerous studies have demonstrated that thermal processing effectively deactivates myrosinase and decreases GLS levels in rapeseed feeds, thus improving their nutritional value for monogastric animals, including poultry [3,22,23]. According to Zentek and Goodarzii-Boorojeni [24], hydrothermal processes can reduce the content of many ANFs in poultry feed components, but their effectiveness is determined by the type and intensity of the applied treatment. Lichovníková et al. [25] and Sasyte et al. [26] observed that extruded rapeseed can be incorporated into layer diets without compromising egg production or egg quality, but little is known about other thermal processing methods for improving the nutritional value of layer diets.

Fermentation treatments have also been long used to improve the nutritional value of feed components, including rapeseed, in animal diets [27,28,29,30]. According to Gao et al. [31], fermentation can reduce the levels of aliphatic compounds (by 57.7%), indole GLS (by 97.3%), oligosaccharides (by 73%), lignin and NDF (by 25%), and PA (by 86%) in RSM, depending on processing conditions and inoculum type. However, the efficacy of fermented rapeseed feeds in laying hen nutrition has not been investigated to date. In our previous experiment performed on female turkeys, the dietary inclusion of fermented RC (FRC) at 15% did not compromise the growth performance of birds and had a greater beneficial influence on breast muscle quality than SBM used as the sole protein source [32]. Engberg et al. [33] demonstrated that fermented feeds fed to laying hens positively affected egg weight and eggshell quality.

In light of previous findings, the research hypothesis postulates that the partial replacement of SBM with RC in laying hen diets can improve productivity, and the antioxidant status and quality of eggs. Therefore, the aim of this study was to determine the effect of raw RC (RRC), hydrobarothermally-treated RC (HRC) and FRC added at 20% to layer diets on laying performance, egg traits, and the FA profile and antioxidant status of egg yolks.

## 2. Materials and Methods

The trial with laying hens was conducted at the Animal Research Laboratory (Department of Poultry Science and Apiculture, University of Warmia and Mazury in Olsztyn, Poland) in accordance with EU Directive 2010/63/EU on the protection of animals used for scientific purposes [34].

### 2.1. Preparation of Hydrobarothermally-Treated Rapeseed Cake (HRC) and Fermented Rapeseed Cake (FRC)

Locally produced RRC was purchased from “Agrolok” *Agrolok* in Golub Dobrzyn, Poland. Purified RRC was ground in a roller mill to pass through a 3 mm sieve, and then conditioned at a temperature of 90–95 °C and humidity of around 18%. Next RRC was processed in a hydrothermal reactor in the presence of steam at a temperature of 95–100 °C for 15–20 min. The raw material was expanded at a temperature of 105–110 °C under pressure of 40–60 atm, dried and cooled to the optimal storage temperature and humidity.

Raw RC was fermented as described by Drażbo et al. [35]. RRC was ground and thoroughly mixed with water in a ratio of 1:2. The fermentation process was carried out with the use of commercial 6-phytase enzyme preparation expressed by Pichia pastoris, which was added to RRC in a weight ratio of 1:1000, and thoroughly mixed. Solid-state fermentation was conducted for 24 h at a temperature of 30 °C under anaerobic conditions. Then the enzyme was deactivated at a temperature of 70 °C for 15 min, and the fermented biomass was dried at a temperature of 55 °C. The fermentation process was carried out under patent pending procedure No. 422849.

Rapeseed samples were analyzed in duplicate for the content of dry matter (DM), crude protein (CP), ether extract (EE), crude fat (CF) and crude ash (CA) according to Association of Official Agricultural Chemists AOAC [36] methods 934.01, 976.05, 920.39, 978.10, 942.05 and 984.27, respectively. Gross energy (GE) was determined with an adiabatic bomb calorimeter (KL 12 Mn, Precyzja-Bit PPHU, Bydgoszcz, Poland) standardized with benzoic acid. NSPs were determined by gas−liquid chromatography (constituent neutral sugars) using an SP-2340 column and a Varian CP3380 gas chromatograph (Varian Inc., Palo Alto, CA, USA) and by colorimetry (uronic acids) using a Biochrom Ultrospec 50 (Biochrom Ltd., Cambridge, UK), according to the procedure described by Englyst and Cummings [37,38] with modifications [39]. Uronic acids were determined as described by Scott [40]. Glucosinolates were determined by gas−liquid chromatography as described by Slominski and Campbell [41].

### 2.2. Birds and Housing

The experimental materials comprised 304 Hy-Line Brown laying hens aged 36 weeks, obtained from a local commercial flock. Before the experiment, all birds were weighed individually and were placed in three-tier battery cages (40 × 35 × 60) cm, with a floor slope of 12°) with two hens per cage. The birds were distributed in a completely randomized design to four treatment groups with 38 replicates per treatment and two hens per replicate. The replicates were equally distributed among three cage levels to minimize the cage level effect. Room temperature was maintained at 20 to 22 °C, and the light cycle was set at 16 h of continuous light and 8 h of darkness. The birds had ad libitum access to feed and water throughout the study. The trial started when the hens were 36 weeks old, and lasted for 12 weeks (until 48 weeks of age).

During the experiment, the birds were fed isonitrogenous and isocaloric diets in mash form, with various protein sources. In the control group (C), SBM was the main source of dietary protein, whereas the experimental groups were fed diets containing 20% of RRC, HRC or FRC. All diets were formulated to meet the nutrient requirements of laying hens [42]. The composition of control and experimental diets is presented in Table 1.

### 2.3. Laying Performance

The body weights (BW) of hens were determined at the beginning (36 weeks of age) and at the end (48 weeks of age) of the experiment. Daily records of egg production were kept, and the egg laying rate was expressed as an average hen-day production. Eggs were collected daily, and egg production was expressed based on the number of days (% working days) at 4-week intervals. Individual egg weight was recorded by individual egg weighing per cage every 2 weeks, and it was used to calculate average egg weight. Total egg weight was calculated by multiplying average egg weight by egg production. Feed intake was controlled every 4 weeks, at 40, 44 and 48 weeks of age. Average daily feed intake (ADFI) per bird was calculated based on total feed intake and the number of days in the analyzed period. The feed conversion ratio—FCR (kg feed/kg eggs laid) was estimated based on egg weight and feed intake.

### 2.4. Egg Quality

At the end of the experiment (at 48 weeks of hens’ age), 12 eggs from each group were picked randomly to determine their physicochemical properties. All quality analyses were completed within 24 h of egg collection. The eggs were weighed individually and were broken on the EQM plate measurement stand (Egg Quality Microprocessor, Technical Services & Supplies Ltd., Dunnington, York, UK) to determine albumen height. The average of two measurements of albumen height and egg weight were used to compute the Haugh unit score (HU) [43]. Yolk color intensity was evaluated and scored according to the yolk color fan (DSM, Heerlen, Netherlands) (1, light yellow; 15, orange). Then the yolk was separated from the albumen using a teflon spoon (Tefal, Rumilly, France). The yolk was rolled on a blotting paper towel. Albumen weight was calculated by subtracting the weights of yolk and eggshell from whole egg weight. To determine eggshell weight, eggshells were cleaned of any adhering albumen and membranes, and they were dried at room temperature. The proportions of the yolk, albumen and eggshell were expressed as a percentage of whole egg weight. Eggshell thickness was measured at three different locations (middle, broad and narrow ends) with the use of a digital micrometer gauge (Mitutoyo QuantuMike, Poland Ltd., Wrocław, Poland), and it was expressed as the mean value. Eggshell breaking strength was measured using an egg force reader (Orka Food Technology, Herzliya, Israel).

### 2.5. Cholesterol Concentration and Fatty Acid Composition of the Egg Yolk

The concentration of cholesterol and FA profile were determined in fat extracted from the yolk, according to the method proposed by Folch et al. [44]. Cholesterol was separated from fat after saponification with potassium hyroxide KOH and extraction with ethyl ether, by the modified method of the International Dairy Federation [45]. The samples were analyzed on a PU4600 chromatograph (Unicam, Cambridge, UK) with a flame ionization detector (FID), under the following conditions: glass column length—1 m, inner diameter—4 mm, film thickness—0.25 m, temperature: detector—300 °C, injector—290 °C, column—260 °C, carrier gas—argon, flow rate—50 cm^3^/min, and internal standard—dotriacontane (Sigma, St. Louis, MO, USA). The extracted fat was esterified with a chloroform, methanol, and sulfuric acid mixture, as described by Peisker [46]. The resulting FA methyl esters (FAMEs) were analyzed on a 7890A gas chromatograph (Agilent Technologies Inc., Palo Alto, CA, USA) with a FID and a Supelcowax 10 capillary column (column length—30 m, internal diameter—0.32 mm, film thickness—0.25 m, carrier gas—helium, and temperature: detector—250 °C, injector—230 °C, and column—195 °C). FA peaks were identified by comparing their relative retention times with those of individual FAME reference standards (Supelco) diluted in hexane (1:1, 1:2, 1:3, and 1:4 *v*/*v*).

### 2.6. Antioxidant Status

Homogenates from egg yolks were also analyzed for lipid peroxidation products, i.e., lipid peroxide content according to Gay and Gebicki [47] and malondialdehyde (MDA) content according to Botsoglou et al. [48]. Malondialdehyde was determined based on the reaction of thiobarbituric acid (TBA) with lipid peroxidation end products in an acidic environment and increased temperature to generate a colored adduct. To eliminate the quantities of a complex series of adducts from TBA, the assay was run in the presence of inhibitors, e.g., butylated hydroxytoluene (BHT). The activity of an antioxidant enzyme, superoxide dismutase (SOD), was determined spectrophotometrically in extracted eggs using the adrenaline method [49] at a modified wavelength of 320 nm to achieve greater selectivity of intermediate reaction products. SOD activity was determined by measuring the rate of auto-oxidation of adrenaline at 30 °C based on an increase in absorbance at 320 nm (which corresponds to the increase in the concentrations of various products of adrenaline oxidation). The activity of catalase (CAT) was determined according to Claiborne pp. 283–284 in [49]. The assay consisted of measuring the rate of substrate (hydrogen peroxide) decomposition catalyzed by this enzyme.

### 2.7. Sensory Evaluation

Hard-boiled eggs were subjected to a sensory evaluation. Before the analysis, the eggs were immersed in a water bath and held at a temperature of 100 °C for 15 min. At the completion of the thermal treatment, the eggs were cooled under a stream of cold running water for 15 min. Immediately after cooling, the eggs were evaluated on a five-point hedonic scale where: 5—very high quality, 4—high quality, 3—satisfactory quality, 2—unsatisfactory quality, 1—poor quality. The following sensory quality attributes were evaluated: appearance (including the appearance of the whole egg and its longitudinal cross-section), aroma, albumen texture, yolk texture, taste. The analysis was performed by six trained panelists selected for their sensory sensitivity [50] (The panelists assessed samples in individual compartments. Fluorescent white lights (500 lux) that simulated daylight were installed at a height of approximately 1 m to evenly illuminate the table. Relative air humidity of minimum 60% and temperature of 21 °C were maintained in the panel room.

### 2.8. Statistical Analysis

For a statistical analysis of performance parameters, a cage was considered as a replicate experimental unit. For analyses of egg quality, FA profile, and the antioxidant parameters of yolks, individual eggs were considered as experimental units. One-way ANOVA was performed with the use of Statistica 13.1 software (StatSoft Corp., Cracow, Poland). When a significant treatment effect was noted, Tukey’s post hoc test was applied to determine differences between treatment groups. Data are presented as means with pooled standard error of the mean (SEM) estimates, and the value of *p* ≤ 0.05 was considered statistically significant.

## 3. Results

The effect of hydrobarothermal treatment and fermentation on the concentrations of nutrients and ANFs in RC is presented in Table 2. In comparison with RRC, HRC was characterized by lower concentrations of CF (9.91 vs. 10.80%) and GLS (13.32 vs. 15.35 μmol/g), whereas the content of the remaining components did not change. Fermentation had a more significant influence on the nutritional value of RC; FRC had a higher content of DM (94.57 vs. 88.87%), CP (39.44 vs. 37.74%) and GE (21.86 vs. 21.19 MJ/kg) than RRC, and the lowest concentration of CF (9.29%) of all analyzed rapeseed products. The greatest changes were observed in the content of ANFs in FRC—the concentrations of PP and GLS decreased two-fold (0.833 vs. 1.607%) and nearly 17-fold (0.92 vs. 15.35 µmol/g), respectively, compared with RRC. A minor increase in NSP content was also noted (21.64 vs. 20.48%).

At the end of the experiment, an increase in the BW of hens was noted only in groups HRC and FRC, whereas hens fed HRC had the significantly highest final BW, relative to group C (*p* = 0.037, Table 3). The applied dietary treatments had no effect on egg weight, but the inclusion of 20% RRC in layer diets significantly decreased laying rate and, consequently, egg mass during the entire experiment, compared with group C (*p* = 0.001 and *p* = 0.014, respectively). Although ADFI was comparable in all groups, the FCR was highest in group RRC, and a significant difference was noted relative to group C (*p* = 0.026). The inclusion of HRC and FRC in layer diets had a greater beneficial influence on the above parameters, and the best results were noted in group FRC where the laying rate was comparable with that in group C and significantly higher than in group RRC. The numerical values of egg mass and the FCR were more favorable in groups HRC and FRC, compared with group RRC, but less satisfactory than in group C.

An analysis of the physicochemical properties of eggs revealed that eggshell quality was highest in group C (Table 4). The inclusion of HRC and FRC in layer diets decreased eggshell thickness and strength. Eggshell thickness was significantly lower in group HRC than in group C, and in group FRC than in group RRC (*p* = 0.001). Eggshell strength was lowest in group HRC (*p* = 0.018). Fermentation had a greater beneficial influence on the parameters of albumen quality than hydrobarothermal treatment. Both albumen height and HU values were highest in eggs produced in group FRC, and significant differences were found relative to group HRC (*p* = 0.001). The addition of RC to layer diets, regardless of its form, improved yolk color, and the highest values of yolk color intensity (DSM yolk color fan) were noted in group HRC (*p* = 0.005). The applied dietary treatments had no effect on the percentage content of yolk, albumen and shell in eggs.

Dietary protein sources had a significant effect on the FA profile of yolk lipids (Table 5). The inclusion of RRC, HRC, and FRC in layer diets contributed to an increase (*p* = 0.001) in the concentrations of linoleic acid (C18:2, n-6), linolenic acid (C18:3, n-3) and docosahexaenoic acid (DHA, C22:6, n-3). The addition of HRC led to a decrease in the content of *arachidonic acid* (AA, C20:4, n-6) in egg yolks, compared with the remaining groups (*p* = 0.002). The concentrations of SFAs were highest in egg yolks in group C (*p* = 0.001). The eggs laid by hens receiving RRC, HRC, and FRC were characterized by significantly highest proportions of PUFAs, n-6 PUFAs, and n-3 PUFAs in the total FA pool, compared with group C (*p* = 0.001), and a much more favorable n-6/n-3 PUFA ratio (*p* = 0.001). In all experimental groups fed 20% RC, eggs had a significantly higher content of hypocholesterolemic FAs and a lower content of hypercholesterolemic FAs, relative to group C (*p* = 0.001). Cholesterol concentration in egg yolks was lowest in group HRC (*p* = 0.001)

Indicators of the redox status of egg yolks are presented in Table 6. Irrespective of its form, RC had no effect on the concentrations of MDA and total glutathione (GSH/GSSG) in egg yolks. A significant decrease in SOD activity (*p* = 0.001) and an increase in CAT activity in eggs were observed in groups RRC, HRC, and FRC, and CAT activity was higher in group FRC (*p* = 0.002) than in group C. An analysis of LOOH concentrations revealed that the content of lipid oxidation products increased significantly in the yolks of eggs laid by hens fed RRC (*p* = 0.001). Both hydrobarothermal treatment and fermentation of RC decreased LOOH concentrations in egg yolks, compared with RRC and significant differences were noted in group FRC where LOOH concentrations were comparable with those in group C.

Regardless of its form, RC added to layer diets had no negative effect on the major sensory attributes of eggs, such as general appearance, albumen texture, yolk texture, and, in particular, taste and aroma (Table 7). A statistical analysis of the results of sensory evaluation demonstrated that eggs produced in group FRC received the highest overall score.

## 4. Discussion

Previous research has demonstrated that hydrothermal and fermentation processes can improve the quality of poultry diets by modifying their chemical composition and reducing the content of ANFs in selected feed components [24,51,52]. In the present study, the content of essential nutrients in RC was not affected by hydrobarothermal treatment or fermentation. The small increase in the crude protein content of FRC can be probably attributed to changes in DM content, rather than an actual increase in protein content [28]. The above observation could also explain the small increase in NSP levels in FRC. In turn, significant changes in ANF content were noted in the components of experimental diets. Glucosinolate levels decreased by 2% in HRC, but they were nearly 17 times lower in FRC than in RRC. Vig and Walia [27] and Chiang et al. [28] observed that the decrease in the GLS content of RMS was proportional to fermentation time, and that isothiocyanate concentration decreased by as much as 83% after 30 days of fermentation. The decrease in the content of GLS and their degradation products during fermentation could be attributed to the breakdown of glucose and sulfur molecules by microbial enzymes [10]. In turn, high-pressure thermal processing inactivates myrosinase that contributes to GLS hydrolysis, leading to the formation of ANFs [53]. The content of PP was two times lower in FRC than in RRC and HRC. According to Fazhi et al. [54], microbial phytases can be used to reduce the PA content of rapeseed during fermentation. In the literature, various microorganisms have been analyzed for their ability to produce phytase and reduce the PA levels in RSM during fermentation [55,56].

Diets with increased content of rapeseed products can compromise feed intake in poultry because GLS degradation products have a bitter taste [12]. Raw RC is also high in fiber, which increases satiety and can additionally decrease feed intake [57]. Numerous studies have demonstrated that diets containing more than 100 g of RSM/kg can reduce feed intake and decrease egg weight [58,59,60]. Interestingly, the above relationship was not observed in the current study, where ADFI and egg weight were comparable in all groups. However, the inclusion of RRC in hen diets significantly decreased the laying rate and egg mass, and it compromised the FCR during the entire experiment. Both rapeseed treatments improved the laying rate, and in groups HRC and FRC, all parameters (that deteriorated in group RRC) were comparable to those noted in group C. Similar results were reported by Jeroch et al. [61,62] who observed that the rapeseed content of layer diets can even be increased to 30% without compromising performance when ANF levels are reduced through combined chemical and hydrothermal treatment.

Eggshell quality plays a very important role in the egg industry [63]. In the present study, layer diets containing 20% of raw or processed RC had a negative influence on eggshell thickness and strength. In the experiments conducted by Riyazi et al. [64], Świątkiewicz et al. [65] and Zhu et al. [66], the incorporation of rapeseed products into layer diets did not affect eggshell quality, whereas Sasyte et al. [26] reported that even small amounts of extruded rapeseed decreased eggshell thickness. Interestingly, in the present study, RRC produced more desirable effects than HRC or FRC, since despite lower numerical values of the analyzed parameters, the results noted in group RRC were statistically comparable to those noted in group C. This was an unexpected outcome because the application of FRC, which was far less abundant in PP than other experimental feed components, should have improved eggshell quality. According to Skřivan et al. [67], high dietary levels of PP decrease phosphorus utilization by laying hens, which can compromise eggshell quality. In turn, FRC improved the parameters of albumen quality. The eggs produced in group FRC were characterized by the highest albumen quality, determined based on albumen height and HU values, and the noted differences were significant relative to group HRC. The above can probably be attributed to a much lower content of GLS in FRC, compared with RRC and HRC. According to Zhu et al. [66], GLS metabolites can inhibit ovalbumin synthesis, decrease albumen height and HU values. However, studies examining the influence of rapeseed products on albumen quality produced ambiguous results. In the work of Lichovníková et al. [68] and Riyazi et al. [64], the incorporation of RSM into layer diets did not affect HU values, whereas in a study by Najib and Al.-Khateeb [59], HU values increased with a rise in the proportion of canola meal in layer diets. However, the effect of treated rapeseed feeds on the above parameters has not been investigated to date. In the present study, the inclusion of rapeseed products in layer diets increased yolk color intensity, which corroborates the findings of other authors [59,60,66]. Treated RC, in particular HRC, delivered the most satisfactory results, but further research is needed to confirm this relationship.

The FA profile of eggs is an important quality attribute for consumers, and efforts are being made to modify layer diets and further improve the FA profile of eggs [69]. Egg yolk PUFAs that deliver the greatest health benefits include linoleic acid (C18:2 n-6, a substrate for the biosynthesis of other long-chain FAs), linolenic acid (C18:3 n-3), EPA (C20:5 n-3), and DHA (C22:6 n-3) [70]. Most of these FAs belong to the family of n-3 PUFAs that contribute to cardiovascular health, brain development and cognitive functioning, and reduce the risk of cancer, autoimmune diseases and diabetes [71,72]. These PUFAs are not synthesized by the body and must be supplied in the diet. In the current study, none of the applied treatments influenced the FA profile of egg yolks relative to group C, whereas all diets containing RRC, HRC, or FRC improved the FA profile of egg yolks relative to group C by increasing the content of linoleic acid, linolenic acid, and DHA, and decreasing the n-6/n-3 PUFA ratio. Buckiuniene et al. [73] reported that an increase in the concentrations of n-3 PUFAs decreased the n-6/n-3 PUFA ratio and enhanced the FA profile of egg yolks. According to the World Health Organization (WHO) recommendations for dietary fat intake, the optimal n-6/n-3 PUFA ratio in the human diet is 4:1 [74]. Kaczmarek et al. [75] observed that RC, a rich source of n-3 PUFAs (including linoleic and linoleic acids) with an n-6/n-3 PUFA ratio of around 2, delivers greater health benefits for monogastric animals than other feeds, which undoubtedly affected the results of the present study. The beneficial effects of rapeseed oil on the FA profile of chicken eggs have been confirmed by many researchers [5,76,77]. However, the influence of fermented or hydrobarothermally-treated RC on the FA profile of chicken eggs has never been examined in the literature. It should be noted that despite the absence of significant differences, egg yolks in group FRC were characterized by the numerically highest content of n-3 PUFAs and the most desirable n-6/n-3 PUFA ratio.

An analysis investigating the effects of various RRC treatments on the cholesterol content of eggs produced rather surprising results. Panaite et al. [78] demonstrated that diets rich in PUFAs can effectively reduce egg cholesterol levels. In the present study, the eggs produced in groups RRC, HRC, and FRC were characterized by the significantly lowest levels of hypercholesterolemic FAs and the highest levels of hypocholesterolemic Fas, which is why egg cholesterol levels were expected to decrease in all experimental groups. Despite the above, egg cholesterol levels decreased significantly only in group HRC, which is difficult to explain. It can only be speculated that lower egg cholesterol levels in group HRC were associated with the metabolism of carotenoids that were easily absorbed, transferred to the egg yolk and significantly improved yolk color. According to Yeum and Russel [79], diets rich in carotenoids decrease cholesterol levels in the blood serum and, probably, in egg yolks.

As previously noted, the inclusion of RC in layer diets increased the content of n-3 PUFAs in egg yolks. However, egg yolks are abundant in lipids, and their susceptibility to peroxidation may increase when layers are fed diets rich in PUFAs [80]. This process leads to the production of MDA, the end product of lipid peroxidation, and the MDA content of tissues indirectly reflects the degree of lipid peroxidation by reactive oxygen species (ROS) [81]. In the current study, the MDA content of egg yolks was comparable in all experimental groups, whereas SOD activity decreased significantly in all groups fed RC, and an increase was observed in CAT activity, in particular in group FRC. Superoxide dismutase and CAT are the main antioxidant enzymes that scavenge ROS via a chain reaction [82]. The MDA content of eggs remained stable, which suggests that dietary modifications did not induce oxidative processes. The observed changes in the activity of antioxidant enzymes could also indicate that RC, in particular FRC, enhances the protective potential of antioxidant enzymes, as demonstrated by the lowest MDA levels and increased CAT activity. According to Ognik and Krauze [83], SOD and CAT activity can be increased or decreased through the addition of feed additives and components with antioxidant properties, but the direction of changes in enzyme activity can be determined by the initial redox potential.

Numerous researchers have demonstrated that the addition of rapeseed products to layer diets can compromise the sensory attributes of eggs, in particular by imparting a fishy taint to eggs [84,85,86]. In the present study, the inclusion of 20% RRC and HRC in layer diets did not affect the taste, aroma, or texture of eggs. Interestingly, FRC significantly improved the above attributes in comparison with control group eggs. In the work of Lichovníková et al. [68] and Świątkiewicz et al. [87], these attributes were already compromised after the addition of 8% untreated rapeseed and rapeseed expeller cake to layer diets, which was attributed to the presence of TMA in the eggs laid by brown hens. The above observation was not confirmed in the current study, and the inclusion of FRC in layer diets led to the production of eggs with the highest sensory quality. These differences could be explained by lower GLS content and, consequently, lower levels of goitrin which inhibits the oxidation of TMA to odorless compounds.

## 5. Conclusions

Hydrobarothermal treatment and fermentation decreased the GLS content of RC, and fermentation reduced PP concentration. As a result, HRC and FRC exerted a greater beneficial influence on the laying performance of hens than raw RRC. RRC, HRC, and FRC can be included at 20% in layer diets because they do not compromise the sensory quality of eggs and have a beneficial influence on the FA profile and antioxidant potential of egg yolks. The use of FRC is recommended because it contributes to the highest laying performance, superior albumen quality, and the highest sensory quality of eggs, relative to RRC and HRC.

## Figures and Tables

**Table 1:** Ingredient composition and nutritional value of experimental diets (%).

Ingredient	Diet			
	C	RRC	HRC	FRC
Wheat	49.756	36.972	36.972	36.972
Maize	20.000	20.000	20.000	20.000
Soybean meal	17.273	7.700	7.700	7.700
Rapeseed cake	-	20	-	-
Hydrobarothermally-treated rapeseed cake	-	-	20	-
Fermented rapeseed cake	-	-	-	20
Soybean oil	1.144	3.842	3.842	3.842
Sodium chloride	0.370	0.372	0.372	0.372
Limestone	9.537	9.319	9.319	9.319
Monocalcium phosphate	1.153	1.123	1.123	1.123
L-Lysine HCL ^1^	0.096	0.079	0.079	0.079
DL-Methionine	0.172	0.092	0.092	0.092
Vitamin-mineral premix ^2^	0.500	0.500	0.500	0.500
Analyzed nutrients				
Crude protein	17.0	16.9	17.1	17.2
Crude fat	3.1	7.1	7.0	7.1
Calculated nutritional value ^3^				
AME, kcal/kg	2700	2700	2700	2700
Crude fiber	2.358	4.765	4.765	4.765
Lysine	0.790	0.790	0.790	0.790
Arginine	0.961	0.936	0.936	0.936
Methionine	0.412	0.371	0.371	0.371
Methionine + Cysteine	0.720	0.720	0.720	0.720
Threonine	0.555	0.601	0.601	0.601
Tryptophan	0.189	0.189	0.189	0.189
Calcium	3.900	3.900	3.900	3.900
Available phosphorus	0.400	0.400	0.400	0.400

^1^ Lysine hydrochloride,^2^ Supplied the following per kilogram of feed: 8000 IU vit A, 2500 IU vit D3, 20 mg vit E, 1.0 mg vit K_3_, 1.5 mg vit B_1_, 4 mg vit B_2_, 1.0 mg vit B_6_, 0.02 mg vit B_12_, 0.1 mg biotin, 6.0 mg pantothenic acid, 65.0 mg Mn from manganese oxide, 52 mg zinc from zinc oxide, 45.0 mg I from ethylene diamine dihydroiodide, 0.15 mg Se from sodium selenite, 6 mg Cu. ^3^ Calculated according to Polish Feedstuff Analysis Tables [42].

**Table 2:** Nutrient composition of rapeseed products and the presence of antinutritional factors (%, dry matter basis).

Content	RRC	HRC	FRC
Dry matter	88.87	88.45	94.57
Crude protein	37.74	37.02	39.44
Crude fat	10.80	9.91	9.29
Gross energy, MJ/kg	21.19	21.41	21.86
Phytate phosphorus	1.607	1.684	0.833
Non-starch polysaccharides ^1^	20.48	20.10	21.64
Glucosinolates (μmol/g) ^2^	15.35	13.32	0.92

^1^ Including rhamnose, arabinose, xylose, mannose, galactose, glucose, and uronic acids. ^2^ Including gluconapin, glucobrassicinapin, progoitrin, glucobrassicin, hydroxyglucobrassicin, and neoglucobrassicin.

**Table 3:** Performance of laying hens fed raw (RRC), hydrobarothermally-treated (HRC) and fermented (FRC) rapeseed cake.

Item	Diet				SEM	*p*-Value
	C	RRC	HRC	FRC		
Initial body weight, kg	2.079	2.040	2.040	2.052	0.014	0.763
Final body weight, kg	1.997 ^b^	2.019 ^ab^	2.096 ^a^	2.054 ^ab^	0.013	0.037
Laying rate, %	96.2 ^a^	92.9 ^b^	94.5 ^ab^	95.4 ^a^	0.285	0.001
Egg weight (g)	61.9	61.5	60.7	61.3	0.243	0.325
Egg mass (kg/hen)	4.95 ^a^	4.76 ^b^	4.81 ^ab^	4.84 ^ab^	0.022	0.014
Average daily feed intake (g/hen/day)	112.6	113.2	112.2	113.3	0.252	0.374
Feed conversion ratio (kg/kg eggs)	1.899 ^b^	1.980 ^a^	1.959 ^ab^	1.952 ^ab^	0.009	0.026

^a,b^ Means within the same column with different superscripts differ significantly (*p* < 0.05).

**Table 4:** Effect of raw (RRC), hydrobarothermally-treated (HRC) and fermented (FRC) rapeseed cake on the physicochemical properties of eggs ^1^.

Item	Diet				SEM	*p*-Value
	C	RRC	HRC	FRC		
Eggshell strength (kg)	4.05 ^a^	3.78 ^ab^	3.30 ^b^	3.70 ^ab^	0.086	0.018
Eggshell thickness, mm	0.314 ^a^	0.306 ^ab^	0.288 ^bc^	0.279 ^c^	0.003	0.001
Yolk color	2.42 ^b^	2.92 ^ab^	3.50 ^a^	3.17 ^ab^	0.115	0.005
Albumen height, mm	7.39 ^ab^	7.29 ^bc^	6.43 ^c^	8.22 ^a^	0.146	0.001
Haugh units	84.0 ^a^	83.7 ^ab^	77.6 ^b^	89.4 ^a^	0.985	0.001
Yolk content, %	25.8	25.9	25.3	25.4	0.758	0.229
Albumen content, %	64.9	65.0	65.8	65.7	0.265	0.505
Shell content, %	9.3	9.1	8.9	8.9	0.087	0.247

^1^ Data representing mean values of 12 eggs per treatment. ^a,b,c^ Means within the same column with different superscripts differ significantly (*p* < 0.05).

**Table 5:** Effect of raw (RRC), hydrobarothermally-treated (HRC) and fermented (FRC) rapeseed cake on the fatty acid composition (% of total fatty acid content) and cholesterol content of yolk lipids (%) ^1^.

Item	Diet				SEM	*p*-Value
	C	RRC	HRC	FRC		
C14:0	0.290 ^a^	0.230 ^b^	0.233 ^b^	0.218 ^b^	0.006	0.001
C14:1	0.060 ^a^	0.024 ^b^	0.022 ^b^	0.021 ^b^	0.003	0.001
C15:0	0.063 ^ab^	0.061 ^ab^	0.057 ^b^	0.069 ^a^	0.002	0.040
C16:0	25.4 ^a^	21.9 ^b^	21.5 ^b^	20.8 ^b^	0.340	0.001
C16:1	3.17 ^a^	1.73 ^b^	1.75 ^b^	1.60 ^b^	0.125	0.001
C17:0	0.167 ^c^	0.211 ^b^	0.216 ^b^	0.245 ^a^	0.005	0.001
C17:1	0.129	0.136	0.128	0.145	0.002	0.468
C18:0	8.13	8.54	8.05	8.45	0.086	0.120
C18:1	42.1	41.4	41.8	41.7	0.208	0.706
C18:2 (n-6)	16.2 ^b^	20.5 ^a^	21.2 ^a^	21.3 ^a^	0.451	0.001
C18:3 (n-6)	0.221 ^b^	0.252 ^a^	0.232 ^ab^	0.255 ^a^	0.004	0.006
C18:3 (n-3)	0.782 ^b^	1.366 ^a^	1.488 ^a^	1.465 ^a^	0.060	0.001
C20:1	0.223 ^a^	0.190 ^b^	0.189 ^b^	0.210 ^ab^	0.004	0.006
C20:2 (n-6)	0.134	0.157	0.167	0.168	0.005	0.096
C20:3 (n-6)	0.141	0.145	0.142	0.142	0.003	0.945
C20:4 (n-6)	1.79 ^a^	1.81 ^a^	1.63 ^b^	1.83 ^a^	0.022	0.002
C22:6 (n-3)	0.912 ^c^	1.326 ^ab^	1.166 ^b^	1.410 ^a^	0.038	0.001
SFAs	34.1 ^a^	30.9 ^b^	30.0 ^b^	29.7 ^b^	0.435	0.001
MUFAs	45.7 ^a^	43.5 ^b^	43.9 ^ab^	43.7 ^b^	0.271	0.010
PUFAs	20.2 ^b^	25.6 ^a^	26.0 ^a^	26.6 ^a^	0.539	0.001
n-3 PUFAs	1.69 ^b^	2.69 ^a^	2.65 ^a^	2.87 ^a^	0.091	0.001
n-6 PUFAs	18.5 ^b^	22.9 ^a^	23.4 ^a^	23.7 ^a^	0.455	0.001
n-6/n-3 PUFA ratio	11.52 ^a^	8.52 ^b^	8.81 ^b^	8.26 ^b^	0.267	0.001
Hypercholesterolemic FAs	26.0 ^a^	22.4 ^b^	22.0 ^b^	21.3 ^b^	0.340	0.001
Hypocholesterolemic FAs	74.0 ^b^	77.6 ^a^	78.0 ^a^	78.7 ^a^	0.340	0.001
Cholesterol	21.0 ^a^	21.4 ^a^	17.0 ^b^	21.8 ^a^	0.522	0.001

^1^ Data representing mean values of 10 eggs per treatment. ^a,b,c^ Means within the same column with different superscripts differ significantly (*p* < 0.05); SFAs—saturated fatty acids, MUFAs—monounsaturated fatty acids, PUFAs—polyunsaturated fatty acids.

**Table 6:** Redox parameters of egg yolks sampled from layers fed a control diet (C), and diets containing raw (RRC), hydrobarothermally-treated (HRC) or fermented rapeseed cake (FRC) ^1^.

Item	Diet				SEM	*p*-Value
	C	RRC	HRC	FRC		
SOD, U/g	123.7 ^a^	99.8 ^b^	98.5 ^b^	104.7 ^b^	2.252	0.001
CAT, U/g	29.7 ^b^	32.9 ^b^	37.0 ^ab^	42.3 ^a^	1.295	0.002
MDA, µmol/kg	7.38	7.67	7.61	7.66	0.148	0.900
LOOH, µmol/L	5.14 ^b^	6.51 ^a^	5.61 ^ab^	5.12 ^b^	0.151	0.001
GSH + GSSG, µmol/kg	4.01	4.06	4.05	4.59	0.142	0.443

^1^ Data representing mean values of 10 eggs per treatment. ^a,b^ Means within the same column with different superscripts differ significantly (*p* < 0.05); SOD, superoxide dismutase; CAT, catalase; MDA, malondialdehyde; LOOH, lipid peroxides; GSH + GSSG, total glutathione.

**Table 7:** Average intensity values of egg sensory attributes ^1^.

Item	Diet				SEM	*p*-Value
	C	RRC	HRC	FRC		
General appearance	3.65 ^b^	3.80 ^b^	3.70 ^b^	4.25 ^a^	0.065	0.002
Aroma	3.75 ^b^	3.75 ^b^	3.75 ^b^	4.15 ^a^	0.057	0.020
Albumen texture	3.80 ^ab^	3.80 ^ab^	3.65 ^b^	4.25 ^a^	0.069	0.008
Yolk texture	3.80 ^b^	3.70 ^b^	3.80 ^b^	4.30 ^a^	0.067	0.004
Taste	3.60 ^b^	3.65 ^b^	3.65 ^b^	4.35 ^a^	0.071	0.001

^1^ Data representing mean values of 10 eggs per treatment. ^a,b^ Means within the same column with different superscripts differ significantly (*p* < 0.05).
